# Changes in Serum Cystatin C Levels and the Associations With Cognitive Function in Alzheimer's Disease Patients

**DOI:** 10.3389/fnagi.2021.790939

**Published:** 2022-01-28

**Authors:** Xueping Chen, Yan Huang, Ting Bao, Fu Jia, Ruwei Ou, Qianqian Wei, Yongping Chen, Jiao Liu, Jing Yang, Huifang Shang

**Affiliations:** West China Hospital, Sichuan University, Chengdu, China

**Keywords:** neurodegenerative diseases, Alzheimer's disease, cystatin C, cognition, association analysis

## Abstract

**Background and Objective:**

Cystatin C is indicated to be involved in the pathogenesis of Alzheimer's disease (AD) and cognitive impairment. Our objective is to examine the serum Cystatin C levels, and to clarify the correlations between serum Cystatin C and cognitive performance in Chinese AD patients.

**Methods:**

The serum Cystatin C concentrations in AD patients and age, sex, and body mass index (BMI) matched-healthy controls were measured. The cognitive functions of the AD patients were evaluated by using the Mini-mental State Examination (MMSE) and the Montreal Cognitive Assessment (MoCA). The severity of dementia was determined with clinical dementia rating (CDR).

**Results:**

A total of 463 AD patients and 1,389 matched healthy subjects were included. AD patients had higher serum Cystatin C than healthy controls. Serum cystatin C levels were correlated with MoCA scores in AD patients. In an ordinal logistic regression model, AD patients with higher serum cystatin C levels had increased odds of severe cognitive dysfunction.

**Conclusion:**

Our study suggested that AD patients had higher levels of serum cystatin C than age/sex/BMI-matched normal control subjects. Higher serum cystatin C may be associated with worse cognitive performance, but more studies are required to verify such association.

## Introduction

Cystatin C is highly expressed in the brain and cerebrospinal fluid (CSF) (Löfberg and Grubb, 1979), and it is indicated to play many roles in the risk and pathobiology for Alzheimer's disease (AD) (Taupin et al., 2000; Palmer et al., 2001; Gauthier et al., 2011). Genetically, the CST3 B haplotype of cystatin C was considered to be a risk factor for AD, frontotemporal dementia (FTD), and Lewy body dementia (LBD) (Finckh et al., 2000; Bertram et al., 2007; Maetzler et al., 2010; Hua et al., 2012). Clinically, in patients with FTD and LBD, a reduction of CSF cystatin C was found, and it was associated with an anticipation of dementia onset (Rüetschi et al., 2005; Sundelöf et al., 2008; Maetzler et al., 2010).

Specifically, in patients with AD, cystatin C levels in CSF were found to be reduced, compared to individuals without dementia (Simonsen et al., 2007; Hansson et al., 2009; Zhong et al., 2013), and CSF cystatin C levels were positively correlated with tau and Aβ levels (Sundelöf et al., 2010; Zhong et al., 2013). However, another study found increased cystatin C levels in CSF of AD patients compared to those in controls (Carrette et al., 2003). In plasma, a study found that AD patients had higher plasma cystatin C levels than healthy control subjects (Wang R. et al., 2017). The association of serum cystatin C with risk of mild cognitive impairment (MCI) or dementia was inconsistent. The Health ABC Study showed that high levels of serum cystatin C increased the risk of cognitive impairment and individuals with higher levels of cystatin C had poorer performance on cognitive examinations (Yaffe et al., 2008). In contrast, the Uppsala Longitudinal Study found that high levels of serum cystatin C were related to a decreased risk of AD in men aged between 70 and 77 years-old (Sundelöf et al., 2008), and a study also found that MCI patients with higher serum cystatin C levels remained stable, without developing to dementia (Romero-Sevilla et al., 2018). Moreover, the Osteoporotic Fractures study reported a *U*-shape association between serum cystatin C and cognitive impairment in elderly women, but the association no longer existed after adjusting for covariates (Slinin et al., 2015). However, there is no study focusing on the association of serum cystatin C levels with cognitive performance in AD patients.

In this study we are aiming to examine the levels of serum cystatin C in AD patients and compare to those in matched healthy subjects, and trying to determine whether serum cystatin C levels are associated with the severity of the dementia.

## Materials and Methods

### Patients and Ethics Statement

This study was performed at the Department of Neurology, Sichuan University West China Hospital, Chengdu, China. From Jan 2014 to Dec 2019, a total of 463 patients with probable AD were enrolled. The diagnosis of AD was based on the criteria issued by the National Institute of Neurological and Communicative Disorders and Stroke-Alzheimer's Disease and Related Disorders Association (NINCDS-ADRDA) The individual, semistructured interviews were conducted with participants and their close informants. Demographic data, including sociodemographic characteristics, lifestyle, medical history, current medications, and family history, were collected. Clinical data related to cognitive impairments, including time of onset, possible triggers, course of condition, impact on daily activities, changes in mood or behavior, and treatment and its effects, were also recored. Last, standardized general and neurological examinations were performed. The diagnoses were made at the end of each interviews according to the NINCDS-ADRDA criteria (McKhann et al., 1984). AD patients participated in the standardized assessments, including the Mini-mental State Examination (MMSE), the Montreal Cognitive Assessment (MoCA), and magnetic resonance imaging (MRI). AD patients with MMSE score higher than 25 were excluded. A total of 1,389 healthy controls (HCs) were also recruited from the Medical Examination Centre of West China Hospital, and they were 3:1 and age/gender/body mass index (BMI)-matched to the AD patients. The HCs also received MMSE and MoCA evaluation. Since MMSE has shown not to be adequate in detecting MCI and clinical signs of dementia, and MoCA is superior to MMSE in the identification of MCI (Pinto et al., 2019), HCs with MMSE score higher than 25 and MoCA sore higher than 22 were included in the present study. Participants, including both AD patients and HCs, who were diagnosed with vascular dementia (VaD), cardiopathy, hypertension, diabetes mellitus, and renal dysfunction, were also excluded. All participants underwent hematological examinations considered to be part of the diagnostic workshop. Peripheral blood samples from the cubital vein were acquired from each AD patient and HCs. Samples were taken by venipuncture, performed between 9:00 and 11:00 a.m., after fasting from midnight. The blood specimens were left at room temperature for 30 min to clot and centrifuged for 10 min at 1,200 g. The cystatin C levels were measured by the automated particle-enhanced immunoturbidimetric method, and the measurement were completed in the Olympus AU5400 analyzer (Olympus, Tokyo, Japan) using the manufacturer's reagents and according to the manufacturer's instructions. The quality of all analyses was assured by appropriate quality control. The kidney function was evaluated using the estimated glomerular filtration rate (eGFR); the calculation of eGFR was completed using the abbreviated Modification of Diet in Renal Disease (MDRD) formula, recommended by K/DOQI as the preferred equation for eGFR (Lameire et al., 2006). When eGFR was 60/mL/min/1.73 m^2^ or less, an impaired renal function was considered. DNA was isolated from blood cells. Samples were amplified by polymerase chain reaction (ABI 7500 FAST, Applied Biosystem, Thermofisher, Waltham, USA). APOE haplotypes were determined according to the manufacturer's instruction with the use of ViennaLab ApoE Strip Assay (Memorigen, Xiamen, China).

This study was approved by the Ethical Committee of West China Hospital of Sichuan University. All AD patients and control subjects gave their written informed consent to participate in the investigation.

### Clinical Evaluation

The following variables were collected: age of onset, sex, BMI, education level, frequent physical activity, smoke, and alcohol consumption. The Clinical Dementia Rating (CDR) is an informant-based global assessment scale with established reliability and validity, and it is utilized as a severity-ranking scale in AD patients in the present study. The cognitive performance was rated in six domains: memory, orientation, judgment and problem solving, community affairs, home and hobbies, and personal care. Each domain is rated according to one of five levels of impairment, and the cognitive dysfunction was defined as follow: mild (CDR = 0.5 or 1), moderate (CDR = 2) and severe (CDR = 3).

### Statistical Analysis

Comparisons of continuous variables between two groups were made using Student's *t*-test. The Kolmogorov-Smirnov test was applied in all continuous variables to verify the presence of normality. A χ2 test was used to compare the categorical variables. One-way ANOVA was performed for three group comparisons of normally-distributed continuous variables. Pearson's non-parametric correlation was applied to investigate the existence of linear association of the serum cystatin C levels and eGFR values to the cognitive performance. The association between cystatin C levels with CDR grading was assessed using ordinal logistic regression model, which was used to predict the CDR stage using serum cystatin C levels in a single model, potential confounders, including age of onset, sex, disease duration, and carriage of the APOE4 allele were allowed in the analyses. This method makes the parallel regression assumption for all variables across the grading of CDR stage. All data were presented in the form of mean ± standard deviation, and they were analyzed using SPSS 17.0. A *p*-value of <0.05 was considered to be statistically significant.

## Results

This cross-sectional study included 463 AD patients [231 males (49.9%) and 232 (50.1%) females], and 1,389 healthy subjects [693 males (49.9%) and 696 females (50.1%)]. The MRI scan supported the diagnosis of the included AD patients, who had medial temporal 146 lobe atrophy, and the Fazekas scale of white matter lesions was <2. The mean age at examination of the AD patients and HCs were 69.00 ± 11.31 and 69.08 ± 11.28 years, respectively. Serum cystatin C levels in AD patients were significantly higher than those in normal subjects (1.034 ± 0.254 vs. 1.010 ± 0.248, *p* = 0.0362). The cognitive performances evaluated by MMSE or MoCA were poorer in AD patients compared to those in HCs (Table 1).

**Table 1:** Demographic and clinical characteristics of patients with AD and healthy controls.

**Characteristics**	**AD (*n* = 463)**	**Controls (*n* = 1,389)**	***p*-value**
Male, *n* (%)	231 (49.9)	693 (49.9)	1
Age at examination, yr	69.00 ± 11.31	69.08 ± 11.28	0.9016
BMI	22.36 ± 3.63	22.58 ± 3.89	0.8321
eGFR (ml/min)	101.13 ± 21.16	104.76 ± 19.53	0.5961
APOE 4 allele carriers (%)	33.48%		
Serum cystatin C (mg/l)	1.034 ± 0.254	1.010 ± 0.248	***P*** **= 0.0362**
MMSE	15.92 ± 7.33	27.18 ± 3.23	***p*** **< 0.001**
MoCA	10.33 ± 6.45	24.98 ± 1.13	***p*** **< 0.001**
Age at onset, yr	67.58 ± 10.12		
Disease duration, mo	17.61		
CDR staging (0.5 or 1/2/3)	148/163/152		
MMSE according to CDR staging	22.88 ± 2.93/15.47 ± 3.29/6.68 ± 3.33		
MoCA according to CDR staging	17.65 ± 4.22/10.19 ± 3.35/5.25 ± 2.85		

*yr, year; mo, month; MMSE, Mini-Mental State Examination; MoCA, Montreal Cognitive Assessment (MoCA); CDR, clinical dementia rating. The meaning of the bold values is P < 0.05*.

In order to determine the association between serum cystatin C levels and cognitive impairment, we stratified the cohort based on the CDR grade (mild CDR = 0.5 or 1, moderate CDR = 2, severe CDR = 3). The one-way ANOVA analyses showed that the serum cystatin C levels were not significantly different among AD patients with different severities of dementia (*p* = 0.6588). In addition, the correlation analyses were utilized to investigate the correlations between serum cystatin C levels and cognitive performance assessed by MMSE and MoCA (Table 2), and a significant correlation between serum cystatin C levels and MoCA scores was identified (r_s_ = −0.1326, *p* = 0.046). However, the correlation analyses showed that serum cystatin C levels were not correlated to the cognitive performance in HCs (MMSE: r_s_ = −0.09827, *p* = 0.1728; MoCA: *r*_s_ = −0.1455, *p* = 0.0798). Serum cystatin C levels was also found to be significantly associated with age at examination in both AD patients and HCs (AD patients: *r*_s_ = 0.4869, *p* < 0.0001; HCs: *r*_s_ = 0.5033, *p* < 0.0001). The ordinal logistic regression stratified by CDR grade was conducted, and the results of the model for predicting CDR grade using cystatin C level showed age at onset, sex, disease duration and presence of *APOE* ε4 allele were not significant risk factors in this model. However, serum cystatin C levels were slightly associated with severity of cognitive impairment (OR = 1.438, 95% CI 0.017–2.858, *p* = 0.047). With regard to the cognitive predictors, AD patients with a 1-point increase in cystatin C levels were 48% more likely to have a higher CDR grade, indicating the AD patients with higher cystatin C levels may have increased odds of severe cognitive dysfunction.

**Table 2:** Correlations of serum cystatin C levels and eGFR values with cognitive performance.

	**Serum cystatin C**		**eGFR values**	
	***r***	***p*-value**	***R***	***p*-value**
MMSE	−0.08484	0.1496	−0.03495	0.6295
MoCA	−0.1326	**0.0460**	−0.1282	0.1230

*The meaning of the bold values is P < 0.05*.

## Discussion

Cystatin C, a cysteine protease inhibitor, is produced by most nucleated cells and present in all body fluids. In the present study, we found that serum cystatin C levels were increased in AD patients than healthy controls, which was supported by some studies (Straface et al., 2005; Wang R. et al., 2017). In contrast, a previous study reported that the plasma cystatin C levels were lower in AD patients than in controls (Chuo et al., 2007), and another studies did not find significant differences in plasma cystatin C levels between AD patients and healthy controls (Kálmán et al., 2000; Ghidoni et al., 2010; Zhong et al., 2013). Plasma cystatin C was also indicated to modulate the clinical expression of cognitive decline; a significant anticipation of the conversion to dementia was observed in MCI subjects, when the detected plasma cystatin C levels were below 1,067 ng/ml (Ghidoni et al., 2010). Such discrepancy may be caused by the differences in sample sizes, characters of participants and genetic backgrounds. To our knowledge, the current study included the largest sample to determine the difference in serum cystatin C levels between AD patients and healthy subjects.

Cystatin C is an inhibitor of cathepsins. Cathepsin D is suggested to be involved in the pathogenesis of AD, and the cathepsin D level is increased in AD patients (Nixon, 2000). The increase of this cathepsin-inhibitory enzyme in AD patients may represent a compensatory activity aimed at counteracting the increased presence of cathepsin D. Cystatin C levels were also increased in response to injury and oxidative stress (Finckh et al., 2000; Nishio et al., 2000). In the central nervous system (CNS), cystatin C can protect neuronal cells from degeneration induced by fibrillar and oligomeric Aβ (Tizon et al., 2010). Co-incubate cystatin C with monomeric Aβ42 can attenuate the formation of Aβ oligomers and protofibrils (Sastre et al., 2004; Selenica et al., 2007), and increase the cystatin C expression can reduce parenchymal Aβ load in CNS (Kaeser et al., 2007; Mi et al., 2007). In addition, the peripheral production of Aβ can be derived from peripheral organs and tissues, including various blood and endothelial cells (Wang J. et al., 2017). The accumulation of Aβ deposits in the peripheral system may stimulate the expression of cystatin C. However, the exact mechanism of increased serum cystatin C in AD patients is not clear. We have to notice that the serum cystatin C is cleared from the circulation by glomerular filtration, and it can be affected by renal function, but the increase of serum cystatin C level in AD patients cannot be simply attributable to the changes of renal function since we did not find any difference in eGFR between AD patients and controls. Furthermore, ANCOVA adjusting for eGFR showed consistent result of significantly higher serum cystatin C levels in AD patients. The findings of the association of serum cystatin C with cognitive impairment were inconsistent: one study did not find any associations in AD patients (Zhong et al., 2013), but another study found a significant correlation between serum cystatin C levels and MMSE scores in female AD patients (Wang R. et al., 2017). In the current study, we found that serum cystatin C levels were negatively associated with cognitive function assessed by MoCA, higher serum cystatin C levels were weakly related to poorer cognitive performance. In addition, we found a significant positive correlation between serum cystatin C and age in both AD patients and healthy subjects. Previous studies also found this association in AD patients (Chuo et al., 2007; Zhong et al., 2013). Therefore, the association between serum cystatin C and age is not disease-specific. Since age is the biggest risk factor for AD (McCartney et al., 2018), we compared the age-at-examination among AD patients with different CDR score, but the differences in age-at-examination were not significant in patients with different level of cognitive dysfunction. We also compared the serum cystatin C levels among AD patients with different severity of dementia, and we did not find any differences in serum cystatin C levels among patients with mild, moderate, or severe dementia evaluated by CDR. However, using ordinal logistic regression models, we found that serum cystatin C levels were slightly associated with cognitive dysfunction; patients with higher cystatin C levels had increased odds of severe cognitive impairment. Similarly, a previous study found that among community-resident elders, those with increased serum cystatin C levels had poorer performance in cognitive tests evaluated by the Modified Mini-Mental State Examination (3MS) and the Digit Symbol Substitution Test (DSST) (Yaffe et al., 2008). Another study also evaluated the cystatin C levels in 193 participants older than 90, and found that higher tertiles of cystatin C was related with poorer global cognition, executive function and visual-spatial ability (Lau et al., 2020). Similarly, the Cardiovascular Health Study Cognition Study reported that high serum levels of cystatin C were related to poorer cognitive performance 6 years later (Riverol et al., 2015). However, the results of previous studies that correlated cystatin C with AD required careful interpretation. First, the association between cystatin C and cognition may underscore the connection between kidney function and cognitive function. Studies have shown that subjects with renal dysfunction had an elevated risk of developing dementia and poorer performance on cognitive function (Seliger et al., 2004; Martens et al., 2017). Cystatin C was found to be associated with cognitive performance in 90+ year-olds; among them nearly 90% had cystatin C levels more than 1.0, indicating that the kidney function is impaired. Further study also gave 308 individuals aged 90 or older a PET scan and obtained the brain indices of Aβ deposition using a statistically defined region of interest (statROI), and found that PET statROI was not correlated with cystatin C and eGFR, indicating an independent association between cognition and chronic kidney disease (CKD), and the CKD-associated cognitive dysfunction largely reflects vascular rather than Aβ pathology (Lau et al., 2021). Second, the correlation between serum cystatin C and cognitive function may not only depend on kidney function. In the present study, no significant differences in eGFR were found among patients with different degrees of dementia stratified by CDR grade, and the eGFR values were not associated with cognition evaluated by MMSE and MoCA. In addition, a previous study also found that elders with high serum cystatin C levels had higher risks of cognitive impairment whether or not they had CKD (Yaffe et al., 2008).

There are some limitations in the present study which should not be ignored: (1) the genetic CST3 genotypes were not considered; (2) the results of the present cannot be extrapolated to other populations since our patients were recruited from a single specialized unit. On the other hand, some of the strengths of our study are the strict inclusion criteria, the examination of APOE genotype, and the fact that the specialized neurologists conducted all the data collections and manifestation assessments.

## Conclusion

In summary, the findings of the current study support the idea that cystatin C plays an essential role in the pathogenesis of AD. Our results suggest AD patients had higher levels of serum cystatin C when compared to age/sex/BMI-matched normal controls, and higher serum cystatin C levels were weakly associated with worse cognitive performance in AD patients. Our findings strengthen the evidence that serum cystatin C may be involved in modulating the clinical expression of cognitive decline. Further longitudinal studies in larger cohorts and distinct ethnic groups will be needed to validate our findings, and to determine the mechanisms underlying this association.

## Data Availability Statement

The original contributions presented in the study are included in the article/supplementary material, further inquiries can be directed to the corresponding author/s.

## Author Contributions

HS conceived this study. YH, TB, RO, QW, YC, JL, and JY did the patient evaluation and data collection. FJ did the statistical analysis. XC and HS wrote the manuscript while all authors revised and discussed to the final edition. All authors contributed to the article and approved the submitted version.

## Funding

This study was supported by grant from National key Research and development program of China (Grant No. 2017YFC09007703), grant from science and technology planning project in Sichuan Province (Grant No. 2020YJ0281), grant from 1·3·5 project for disciplines of excellence West China Hospital Sichuan University (Grant No. ZYJC18038), and the grant from cadres health care project in Sichuan Province (Grant No. 2019-112).

## Conflict of Interest

The authors declare that the research was conducted in the absence of any commercial or financial relationships that could be construed as a potential conflict of interest.

## Publisher's Note

All claims expressed in this article are solely those of the authors and do not necessarily represent those of their affiliated organizations, or those of the publisher, the editors and the reviewers. Any product that may be evaluated in this article, or claim that may be made by its manufacturer, is not guaranteed or endorsed by the publisher.

## References

[B1] Bertram L. McQueen M. B. Mullin K. Blacker D. Tanzi R. E. (2007). Systematic meta-analyses of Alzheimer disease genetic association studies: the AlzGene database. Nat. Genet. 39, 17–23. 10.1038/ng1934 17192785

[B2] Carrette O. Demalte I. Scherl A. Yalkinoglu O. Corthals G. Burkhard P.. (2003). A panel of cerebrospinal fluid potential biomarkers for the diagnosis of Alzheimer's disease. Proteomics 3, 1486–1494. 10.1002/pmic.200300470 12923774

[B3] Chuo L. J. Sheu W. H. Pai M. C. Kuo Y. M. (2007). Genotype and plasma concentration of cystatin C in patients with late-onset Alzheimer disease. Dement. Geriatr. Cogn. Disord. 23, 251–257. 10.1159/000100021 17310123

[B4] Finckh U. von der Kammer H. Velden J. Michel T. Andresen B. Deng A.. (2000). Genetic association of a cystatin C gene polymorphism with late-onset Alzheimer disease. Arch. Neurol. 57, 1579–1583. 10.1001/archneur.57.11.1579 11074789

[B5] Gauthier S. Kaur G. Mi W. Tizon B. Levy E. (2011). Protective mechanisms by cystatin C in neurodegenerative diseases. Front. Biosci. 3, 541–554. 10.2741/s170 21196395 PMC3038625

[B6] Ghidoni R. Benussi L. Glionna M. Desenzani S. Albertini V. Levy E.. (2010). Plasma cystatin C and risk of developing Alzheimer's disease in subjects with mild cognitive impairment. J. Alzheimers Dis. 22, 985–991. 10.3233/JAD-2010-101095 20858959

[B7] Hansson S. F. Andréasson U. Wall M. Skoog I. Andreasen N. Wallin A. (2009). Reduced levels of amyloid-beta-binding proteins in cerebrospinal fluid from Alzheimer's disease patients. J. Alzheimers Dis. 16, 389–397. 10.3233/JAD-2009-0966 19221428

[B8] Hua Y. Zhao H. Lu X. Kong Y. Jin H. (2012). Meta-analysis of the cystatin C(CST3) gene G73A polymorphism and susceptibility to Alzheimer's disease. Int. J. Neurosci. 122, 431–438. 10.3109/00207454.2012.672502 22435454

[B9] Kaeser S. A. Herzig M. C. Coomaraswamy J. Kilger E. Selenica M. L. Winkler D. T.. (2007). Cystatin C modulates cerebral beta-amyloidosis. Nat. Genet. 39, 1437–1439. 10.1038/ng.2007.23 18026102

[B10] Kálmán J. Márki-Zay J. Juhász A. Sántha A. Dux L. Janka Z. (2000). Serum and cerebrospinal fluid cystatin C levels in vascular and Alzheimer's dementia. Acta Neurol. Scand. 101, 279–282. 10.1034/j.1600-0404.2000.101004279.x 10770527

[B11] Lameire N. Adam A. Becker C. R. Davidson C. McCullough P. A. Stacul F.. (2006). Baseline renal function screening. Am J Cardiol. 98, 21K–26K. 10.1016/j.amjcard.2006.01.021 16949377

[B12] Lau W. L. Fisher M. Fletcher E. DeCarli C. Troutt H. Corrada M. M.. (2021). Kidney function is not related to brain amyloid burden on PET imaging in the 90+ Study Cohort. Front. Med. 8, 671945. 10.3389/fmed.2021.671945 34616751 PMC8488112

[B13] Lau W. L. Fisher M. Greenia D. Floriolli D. Fletcher E. Singh B.. (2020). Cystatin, C., cognition, and brain MRI findings in 90+-year-olds. Neurobiol. Aging 93, 78–84. 10.1016/j.neurobiolaging.2020.04.022 32473464 PMC7307913

[B14] Löfberg H. Grubb A. O. (1979). Quantitation of gamma-trace in human biological fluids: indications for production in the central nervous system. Scand. J. Clin. Lab. Invest. 39, 619–626. 10.3109/00365517909108866 119302

[B15] Maetzler W. Schmid B. Synofzik M. Schulte C. Riester K. Huber H.. (2010). The CST3 BB genotype and low cystatin C cerebrospinal fluid levels are associated with dementia in Lewy body disease. J. Alzheimers Dis. 19, 937–942. 10.3233/JAD-2010-1289 20157249

[B16] Martens R. J. Kooman J. P. Stehouwer C. D. Dagnelie P. C. van der Kallen C. J. Koster A.. (2017). Estimated GFR, albuminuria and cognitive performance: The Maastricht Study. Am. J. Kidney Dis. 69, 179–191. 10.1053/j.ajkd.2016.04.017 27291486

[B17] McCartney D. L. Stevenson A. J. Walker R. M. Gibson J. Morris S. W. Campbell A.. (2018). Investigating the relationship between DNA methylation age acceleration and risk factors for Alzheimer's disease. Alzheimers Dement. 10, 429–437. 10.1016/j.dadm.2018.05.006 30167451 PMC6111045

[B18] McKhann G. Drachman D. Folstein M. Katzman R. Price D. Stadlan E. M. (1984). Clinical diagnosis of Alzheimer's disease: report of the NINCDS-ADRDA Work Group under the auspices of Department of Health and Human Services Task Force on Alzheimer's Disease. Neurology 34, 939–944. 10.1212/WNL.34.7.939 6610841

[B19] Mi W. Pawlik M. Sastre M. Jung S. S. Radvinsky D. S. Klein A. M.. (2007). Cystatin C inhibits amyloid-beta deposition in Alzheimer's disease mouse models. Nat. Genet. 39, 1440–1442. 10.1038/ng.2007.29 18026100

[B20] Nishio C. Yoshida K. Nishiyama K. Hatanaka H. Yamada M. (2000). Involvement of cystatin C in oxidative stress-induced apoptosis of cultured rat CNS neurons. Brain Res. 873, 252–262. 10.1016/S0006-8993(00)02540-3 10930551

[B21] Nixon R. A. (2000). A “protease activation cascade” in the pathogenesis of Alzheimer's disease. Ann. N. Y. Acad. Sci. 924:117–131. 10.1111/j.1749-6632.2000.tb05570.x 11193788

[B22] Palmer T. D. Schwartz P. H. Taupin P. Kaspar B. Stein S. A. Gage F. H. (2001). Cell culture. Progenitor cells from human brain after death. Nature 411, 42–43. 10.1038/35075141 11333968

[B23] Pinto T. C. C. Machado L. Bulgacov T. M. Rodrigues-Júnior A. L. Costa M. Ximenes R.. (2019). Is the Montreal Cognitive Assessment (MoCA) screening superior to the Mini-Mental State Examination (MMSE) in the detection of mild cognitive impairment (MCI) and Alzheimer's Disease (AD) in the elderly? Int. Psychogeriatr. 31, 491–504. 10.1017/S1041610218001370 30426911

[B24] Riverol M. Becker J. T. López O. L. Raji C. A. Thompson P. M. Carmichael O. T.. (2015). Relationship between systemic and cerebral vascular disease and brain structure integrity in normal elderly individuals. J. Alzheimers Dis. 44, 319–328. 10.3233/JAD-141077 25213770 PMC4297227

[B25] Romero-Sevilla R. Casado-Naranjo I. Portilla-Cuenca J. C. Duque-de San Juan B. Fuentes J. M. Lopez-Espuela F. (2018). Vascular risk factors and lesions of vascular nature in magnetic resonance as predictors of progression to dementia in patients with mild cognitive impairment. Curr. Alzheimer Res. 15, 671–678. 10.2174/1567205015666180119100840 29357793

[B26] Rüetschi U. Zetterberg H. Podust V. N. Gottfries J. Li S. Hviid Simonsen A.. (2005). Identification of CSF biomarkers for frontotemporal dementia using SELDI-TOF. Exp. Neurol. 196, 273–281. 10.1016/j.expneurol.2005.08.002 16154129

[B27] Sastre M. Calero M. Pawlik M. Mathews P. M. Kumar A. Danilov V.. (2004). Binding of cystatin C to Alzheimer's amyloid beta inhibits *in vitro* amyloid fibril formation. Neurobiol. Aging 25, 1033–1043. 10.1016/j.neurobiolaging.2003.11.006 15212828

[B28] Selenica M. L. Wang X. Ostergaard-Pedersen L. Westlind-Danielsson A. Grubb A. (2007). Cystatin C reduces the *in vitro* formation of soluble Abeta1-42 oligomers and protofibrils. Scand. J. Clin. Lab. Invest. 67, 179–190. 10.1080/00365510601009738 17365997

[B29] Seliger S. L. Siscovick D. S. Stehman-Breen C. O. Gillen D. L. Fitzpatrick A. Bleyer A.. (2004). Moderate renal impairment and risk of dementia among older adults: the Cardiovascular Health Cognition Study. J. Am. Soc. Nephrol. 15, 1904–1911. 10.1097/01.ASN.0000131529.60019.FA 15213280

[B30] Simonsen A. H. McGuire J. Podust V. N. Hagnelius N. O. Nilsson T. K. Kapaki E.. (2007). A novel panel of cerebrospinal fluid biomarkers for the differential diagnosis of Alzheimer's disease versus normal aging and frontotemporal dementia. Dement. Geriatr. Cogn. Disord. 24, 434–440. 10.1159/000110576 17971664

[B31] Slinin Y. Peters K. W. Ishani A. Yaffe K. Fink H. A. Stone K. L.. (2015). Cystatin C and cognitive impairment 10 years later in older women. J. Gerontol. A Biol. Sci. Med. Sci. 70, 771–778. 10.1093/gerona/glu189 25362662 PMC4447803

[B32] Straface E. Matarrese P. Gambardella L. Vona R. Sgadari A. Silveri M. C.. (2005). Oxidative imbalance and cathepsin D changes as peripheral blood biomarkers of Alzheimer disease: a pilot study. FEBS Lett. 579, 2759–2766. 10.1016/j.febslet.2005.03.094 15907478

[B33] Sundelöf J. Arnlöv J. Ingelsson E. Sundström J. Basu S. Zethelius B.. (2008). Serum cystatin C and the risk of Alzheimer disease in elderly men. Neurology 71, 1072–1079. 10.1212/01.wnl.0000326894.40353.93 18824671 PMC2676985

[B34] Sundelöf J. Sundström J. Hansson O. Eriksdotter-Jönhagen M. Giedraitis V. Larsson A.. (2010). Cystatin C levels are positively correlated with both Abeta42 and tau levels in cerebrospinal fluid in persons with Alzheimer's disease, mild cognitive impairment, and healthy controls. J. Alzheimers Dis. 21, 471–478. 10.3233/JAD-2010-091594 20555147

[B35] Taupin P. Ray J. Fischer W. H. Suhr S. T. Hakansson K. Grubb A.. (2000). FGF-2-responsive neural stem cell proliferation requires CCg, a novel autocrine/paracrine cofactor. Neuron 28, 385–397. 10.1016/S0896-6273(00)00119-7 11144350

[B36] Tizon B. Ribe E. M. Mi W. Troy C. M. Levy E. (2010). Cystatin C protects neuronal cells from amyloid-beta-induced toxicity. J. Alzheimers Dis. 19, 885–894. 10.3233/JAD-2010-1291 20157244 PMC2889175

[B37] Wang J. Gu B. J. Masters C. L. Wang Y. J. (2017). A systemic view of Alzheimer disease - insights from amyloid-β metabolism beyond the brain. Nat. Rev. Neurol. 13, 612–623. 10.1038/nrneurol.2017.111 28960209

[B38] Wang R. Chen Z. Fu Y. Wei X. Liao J. Liu X.. (2017). Plasma cystatin C and high-density lipoprotein are important biomarkers of Alzheimer's disease and vascular dementia: a cross-sectional study. Front. Aging Neurosci. 9, 26. 10.3389/fnagi.2017.00026 28223934 PMC5294921

[B39] Yaffe K. Lindquist K. Shlipak M. G. Simonsick E. Fried L. Rosano C.. (2008). Cystatin C as a marker of cognitive function in elders: findings from the health ABC study. Ann. Neurol. 63, 798–802. 10.1002/ana.21383 18496846 PMC2584446

[B40] Zhong X. M. Hou L. Luo X. N. Shi H. S. Hu G. Y. He H. B.. (2013). Alterations of CSF cystatin C levels and their correlations with CSF Aβ40 and Aβ42 levels in patients with Alzheimer's disease, dementia with lewy bodies and the atrophic form of general paresis. PLoS ONE 8, e55328. 10.1371/journal.pone.0055328 23383156 PMC3558470

